# Mr. Keir Hardie and the Miners

**Published:** 1892-06-25

**Authors:** 


### MR. KEIR HARDIE AND THE MINERS.

By The Author of "Wages versus Comfort."

I was away from home when Mr. Keir Hardie's statement regarding the Lanarkshire miners appeared in the *Daily Chronicle* in protest against an account of mine in The Hospital, and I have, therefore, been slower than I could wish in answering his remarks. These remarks I desire, for the honour in which I hold Mr. Keir Hardie's name, to treat with all respect and sincerity, and to confine every word of mine to the limits of the strictest accuracy. Yet I find that already, in my first sentence, I have been inaccurate. For I said that Mr. Hardie had written a protest against my article, whereas, by his own avowal, he has never seen it, but only the incomplete—and, I am forced to add, prejudiced—*resumé* of it given in the *Daily Chronicle* leaderette. This is surely just neither to himself nor me. He accuses me of writing a "grotesque libel" on the miners; the grotesqueness belongs to the *Daily Chronicle*; as for the libel—in face of Mr. Hardie's admission that there are spots in the sun of collier life which he describes, I need not defend myself against that beyond saying that I live in a "spot," and a big black one, that's all. I have spoken of that which I have seen.

Let me, at the risk of being charged with irrelevance and egotism, dispose of the impression which must have resulted from the perusal of the *Daily Chronicle*, that the writer in The Hospital was simply a malicious fault-finder, or an unscrupulous scribbler ready to misrepresent everything on the chance of making smart copy out of it. I have not come on a brief visit to see what can be made out of the miners, either in their interests or their masters'; but circumstances have placed me in a mining district, and I see an amount of misery, dirt, and degradation which surpasses anything I could have conceived. Coming from London, as I did, I imagined that these people must be in the direst poverty, and was utterly surprised—at first incredulous—when I heard of the wages earned by them. Yet I had lived among colliers before, and made acquaintance with a good many. Indeed, I may count as a valued friend one who, sent into the pits when he was only seven years old, emancipated himself by dint of endless labour and self-denial, and is now the proprietor of a sufficiently prosperous book-selling business, against which he complains, however, that it leaves him too little leisure for his favourite pursuits, literature and archæology. But that was in the Lothians, and I have been repeatedly told that the Lothian miners are of a higher type than the west countrymen. Certainly my two years' experience there left me quite unprepared for Lanarkshire.

Yet even there I did not see these spotless curtains, well-washed floors, well-stocked kailyards, and dainty flower beds which Mr. Hardy speaks of; certainly none of these things have I caught a glimpse of in a collier's house in Lanarkshire. I know them well, indeed, all that Mr. Keir Hardie describes —mahogany drawers, china bowls, and all—in ploughmen's houses where, on that 12s. 6d. a week which in his eyes spells starvation, a large family is honourably reared, the clever son sent to college, the clever daughter trained to be a teacher, in nearly every parish in Scotland. How it is done I don't know; I only know that the agricultural classes do it to a very large extent, and that the colliers do it hardly at all. Yet Mr. Keir Hardie speaks of such a clean colliers' village, with well-furnished, well-cared-for houses, "and Brutus is an honourable man." He speaks of having taken Miss Clementina Black to see these clean, well-furnished houses, and her surprise thereat. As he has introduced Miss Black's name, I may do the same. That lady has told me also of the time when "Mr. Keir Hardie took her through his village." The secret of Mr. Hardie's protest lies in that one word "*his*." The village he describes exists—unique, alone. Mr. Keir Hardie has—shall I say created it?—at least fostered and cared for it and its inhabitants, encouraging every germ of good, developing every glimmer of self-respect and the virtues that spring from it, as I would there were a Keir Hardie in every collier village to do. But while I honour Mr. Keir Hardie for his work, I protest against his describing, in the *Daily Chronicle* or elsewhere, the exceptional for the average. One may study agricultural methods and possibilities in a model farm; but no one would think of taking it for a fair specimen of agricultural habits. It leads the way; it is not parallel. I protest against Mr. Keir Hardie offering us his model farm, so nursed and privileged, as a specimen of the ordinary collier village.

I think that Mr. Keir Hardie, indeed, rather spoils the case he fain would prove by his description of the second room, which he rejoices to see becoming more common in the miner's house. So would I rejoice if I saw it put to some more practical use than that of "a veritable *sanctum sanctorum*, sacred to the household deities, and across which no profane foot is allowed to pass save on rare occasions." That is true, and meanwhile, Mr. Keir Hardie, the whole family herd together in those two fixed-in-beds in the kitchen, and the treasured parlour fails of any practical use. We ask employers, in the name of hygiene and morality, for more accommodation for their workers, and this, at the best, is what comes of it. "What do you mean by a parlour?" asked a schoolmistress I know of one of her pupils. "A room you keep locked up," was the reply. I am able to tell the furnishing of one of these treasure houses. It contained a piano, an American organ, a violin, a concertina, and a melodion. I know there were seven musical instruments in all but I cannot remember the other two. Of course I am aware that the posseser of these shows higher taste than the miner is credited with, as a rule. Such a family is an exceptional one, and a hopeful one on the whole, though since one member of the brass band stabbed another in a public house after they had been playing at a flower show, I have had some doubts as to the refining influence of music on all savage breasts.

But in many cases the extra room, and often enough a bed in the common room occupied by the family is let to lodgers—not usually to one, but to two, who occupy it in turn (in Box and Cox fashion), according to their different night or day shifts. Eight-hour shifts are the custom here—not ten or twelve hours, as in the places which Mr. Keir Hardie speaks of, and which I do not know. As to the way in which wages are made up to the sums I stated, I explained this in a recent number of The Hospital, and as I would much prefer that Mr. Keir Hardie would read anything I have said in The Hospital as I said it, and not in the *Daily Chronicle* as it is therein said that I said it, I will not reproduce my words, but refer him to the original. But even according to his own statement that a boy earns only 2s. or 3s. a day, does it not strike him that from ten to fifteen shillings a week—I count only the five days a week that the collier works—is very good pay for the unskilled labour of a boy of twelve or fourteen? With regard to the standard wage, I returned to Lanarkshire only twenty-four hours ago, and have not had time to enquire, but when my article was written it was considerably more than 5s. a day. I hope soon to have statistics quite up to date with regard to the miners' wages. I despair, however, of thereby convincing Mr. Keir Hardie. If I bring up cases of men earning 7s. or 8s. a day will he not say again, as he does in the *Daily Chronicle* of June 7th, "These, as a rule, are the hirelings of the employers, who are paid to promote dissension among their fellows." I cannot but think that in making such a statement, which he does not in any way try to substantiate, Mr. Keir Hardie generalises, I will not say rashly, but wantonly. Has he a right to complain of "the disorganised state of the county of Lanark" if he speaks of certain men, simply because they are earning more than others, as hirelings "paid to promote dissension among their fellows"? Does he expect to found a strong and brotherly organisation by flinging abroad such accusations as this. To set fellow-workers against each other in this fashion is the work of the mere agitator, of the unscrupulous employer and wily manager, if Mr. Keir Hardie likes, but it seems to me to be unworthy of him.

I am accused also of maligning the miner's wife. Mr. Keir Hardie assures me that "no harder worked woman exists anywhere than the collier's wife." Now I know fishermen's wives, and how they look for the bait and dress the lines, and then go out and sell the fish. I know the wives of agricultural labourers, and how they add to the family income by their help in the dairy and by their work in the strawberry gardens and the harvest field (a woman can make half-a-crown to three shillings a day, with extra pay for overtime in the Lanarkshire strawberry fields; but the work is hard and fatiguing). Withal, these women keep a clean house, as clean and well-furnished as that Mr. Keir Hardie describes, "cook, bake, do their own washing, knitting, and mending," are clever at "making down" their own gowns for the lassies, and the guidman's clothes for the laddies, using up the very scraps and cuttings to make warm "rag carpets" to lay before the fire. In short Burns's cottar's wife and Mr. Barrie's Jean's are true pictures of the Scottish woman of the agricultural, fishing, and artisan classes. But of the collier's wife how much of this is true? That she does not go out to work except when her "man" is idle I do not blame her, for the wages of every worker ought to be sufficient to keep his wife and young children, though I always think that a woman who maintains her economic independence obtains thereby a dignity which cannot be bought. But with all her leisure, free to devote to her household cares, I still maintain that she neglects them more than any other woman in any class. Her house—at least, such as I have seen—is dirty; it has not been my lot to meet the white curtains and spotless linen towel. Her children—again I speak of those whom I have seen, not those whom Mr. Keir Hardie knows—are dirty, their clothes hang torn, or are fixed with pins where buttons have fallen off. They look, indeed, as if they knew something of "flyting and skelping." I have seen one poor arm that had been dislocated by the jerk of an impatient mother: on this point my critic and I are at one. Her cooking and washing are such as to baffle description; she has reduced the arts of life to their simplest proportions, and even then leaves them half unfulfilled. At morn or noon or even you may see her sitting on her doorstep, as lazy at noon as at night; as dirty at night as at noon.

Mr. Keir Hardie declares that drunkenness, thriftlessness, and the rest of the evils I touched on are confined to "certain well-defined localities." I suppose that it is my lot to live in a "well-defined locality," for certainly these are the things that prevail here. It is a large locality, and embraces eight or ten mining towns and villages. But it is strange, if my experience is exceptional, that within a week two people should have spoken to me of the article which the *Daily Chronicle* so unhesitatingly disbelieved. With neither of them have I ever spoken on the subject of miners before. One said, "I don't know anything about Lanarkshire, but all you said would be applicable to the miners in South Wales." Said the other: "I could more than parallel your statements by my experience among the Yorkshire miners." It would seem then that miners are pretty much the same all the land over, save only in the village known to Mr. Keir Hardie.

But, indeed, Mr. Keir Hardie practically admits all that I said, but blames the miners' faults on the "low cunning" of employers and managers. I never undertook to say on whom the responsibility lay, though in this connection I might well quote Miss Black's wise words to her fellow-labourers in the field of literature, when she reminded them that the ultimate decision as to the condition of any labourers, and the reward of their labour lay not with the employer, but with the employed. The final question is what the one will accept, not what the other will give. If Mr. Keir Hardie can develope the moral fibre of the collier, his material condition is bound to improve. If I may, most diffidently, say a word on this subject to colliers and their friends, it would be this: Do not think that high wages are the one thing to strive for. That is a lesson you learned easily enough; but your masters learned it too. The strikes by which you mean to force the master's hand often only fill his pocket. Increase of wages has often meant only increase of temptation, and the money which might have enabled you one day to withstand a real injustice has been melted in the vile liquor of the publican that first maddens and then kills you—a far worse waste than Cleopatra's pearls. From within, not from without, must come the impulse that will raise your work to the dignity which, from its necessary nature and for the dangers attending it, it nobly deserves. The thesis which I set out to prove in my original article, and which has not yet been disproved, was that wages and comfort do not go hand in hand. But they might do so. Your children have the chance of good education free; do not take them away from school at the first moment possible, the very moment when their minds are beginning most actively to profit by what they learn, because of the shillings they can add to the weekly income. Your self-denial will be well paid in the end. Cultivate some ideal in your life; the wiliest manager cannot cheat you out of that. Political freedom you have; the State will do its best to secure you from danger and compensate you for injury. But no Act of Parliament can keep you from injuring yourself. The most paternal government can do little to make you sober, moral, and provident if you yourselves shirk all its restrictions. You have heard enough of what other people—masters, ministers, Members of Parliament—should do for you; but you will rise to better and happier things, healthier conditions of labour, the life of self-respect and culture, which is as open to you as to others, only by developing those diviner elements which are lying in your own soul.

